# Supplementary material for: Atlantic water recirculation in the northern Barents Sea affects winter sea ice extent
Source: Nat Commun. 2025 Jun 19;16:5148. doi: 10.1038/s41467-025-59992-9 (PMC12179290; doi:10.1038/s41467-025-59992-9)
Supplement: Supplementary file 1 — Supplementary Information [file 41467_2025_59992_MOESM1_ESM.pdf]

Supporting Information:

Atlantic Water Recirculation in the Northern Barents Sea Affects Winter Sea Ice Extent

Finn Ole Heukamp[1], Claudia Wekerle[1], Torsten Kanzow[1,2], Rebecca McPherson[1], and Till M. Baumann[3,4]

[1]Alfred-Wegener-Institute for Polar and Marine Research, Bremerhaven, Germany.

[2]University of Bremen, Bremen, Germany.

[3]Institute of Marine Research, Bergen, Norway

[4]Bjerknes Centre for Climate Research, Bergen, Norway.

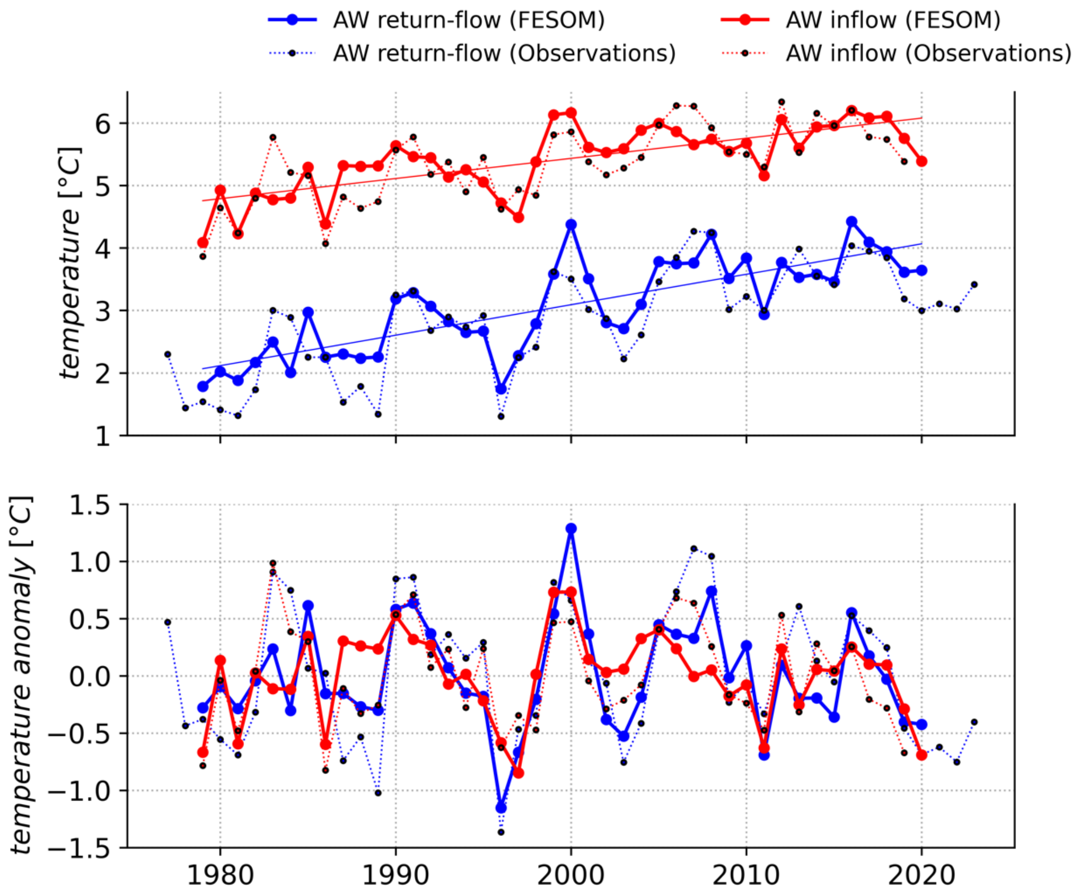

Fig. SI1: Atlantic Water temperature in the Barents Sea Opening. (a) Winter mean (December-March) (a) AW temperature in the central AW inflow of the BSO at 73°N (50-200m) derived from regular CTD stations and FESOM2.1 and in the return-flow of the BSO between 73.67°N and 74.25°N (50 m - bottom, see methods) derived from regular CTD stations and FESOM2.1. (b) As in (a) but with the long-term trend and the mean removed.

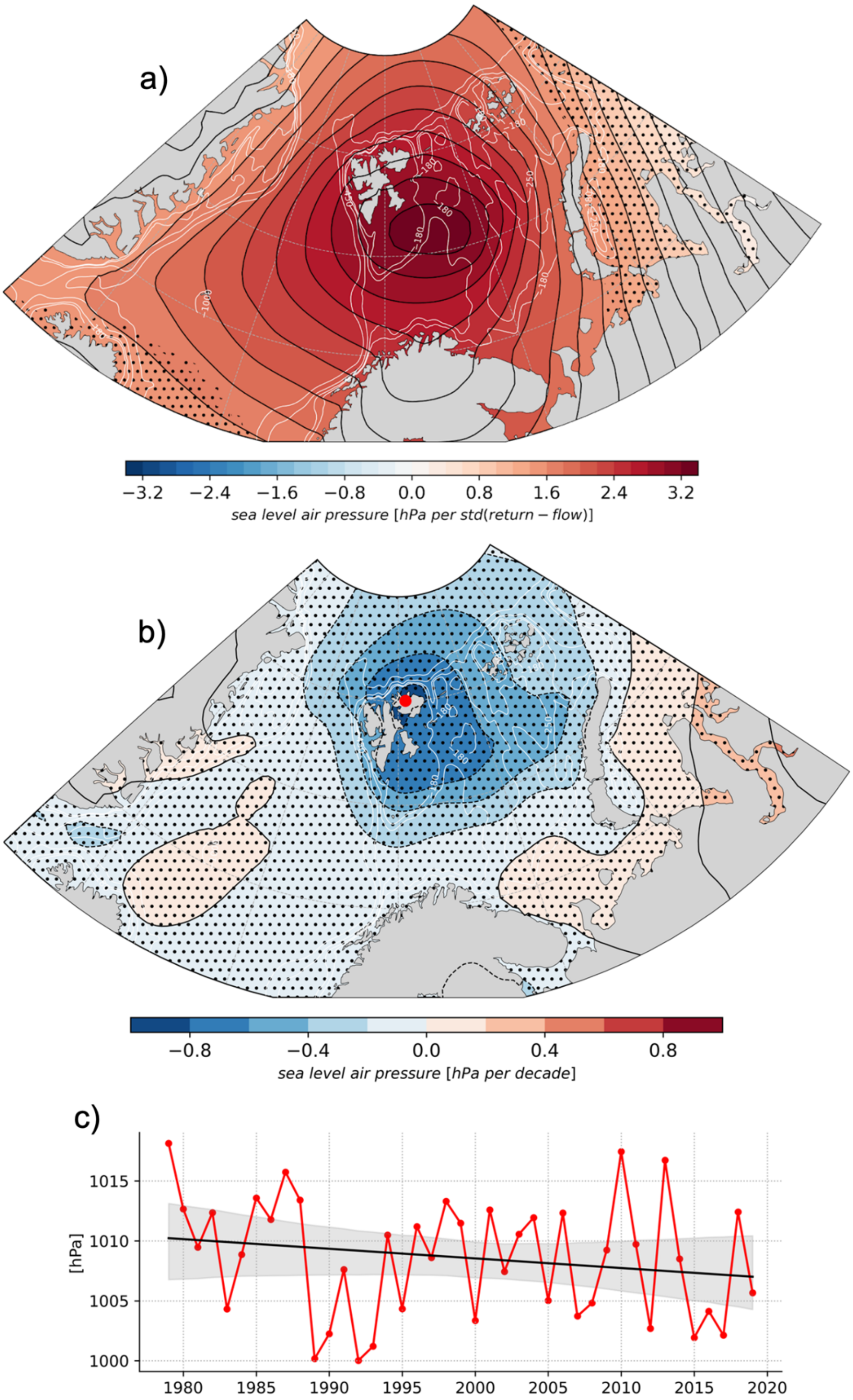

Fig. SI2: Atmospheric driving pattern of the Atlantic Water return-flow. (a) The pattern of anomalous sea level air pressure from a linear regression analysis with the detrended anomalous volume transport of the return-flow as a predictor of the detrended anomalous sea level air pressure (1979-2019). (b) Linear Trend of the sea level air pressure over the northern Barents Sea (1979-2019). Red dot indicates location of most maximum trend in sea level air pressure. Dotted areas in (a) and (b) indicate non-significant regression coefficients (95% confidence). (c) Timeseries of winter mean sea level air pressure at location of maximum trend (red dot in (b)) with its long-term trend and the 95% confidence interval of the regression slope.

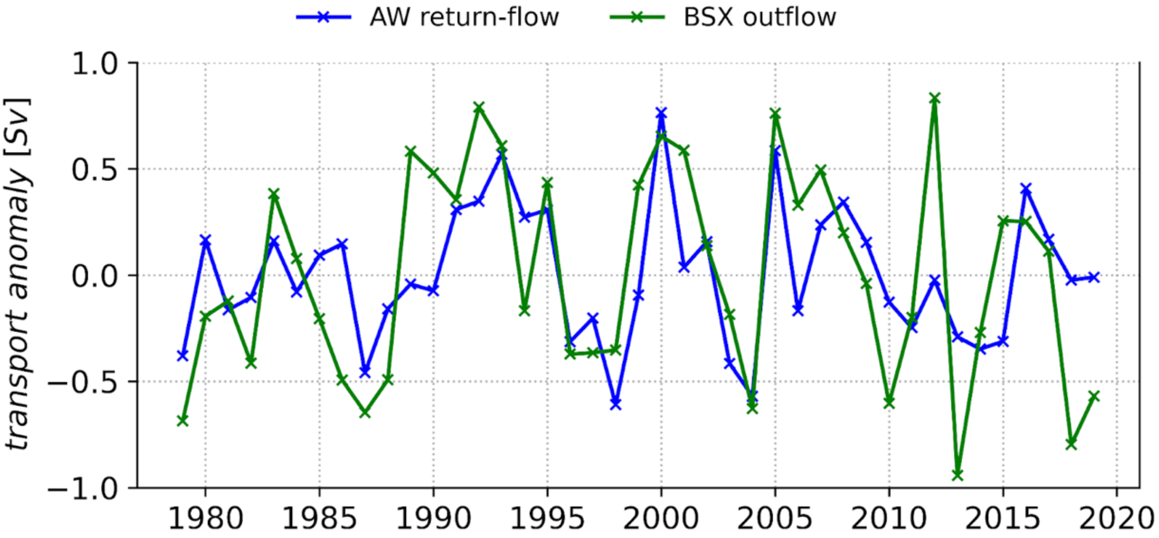

Fig. SI3: Atlantic Water volume transport anomalies on the Barents Sea Opening and Barents Sea Exit. Time-series of the winter mean Atlantic Water volume transport anomalies of the AW return-flow in the BSO and the eastward transport through the BSX. Note that positive return-flow transport anomalies depict decreased transport from the BS into the Nordic Seas whereas positive BSX outflow transport anomalies depict increased transport from the Barents Sea into the Kara Sea.

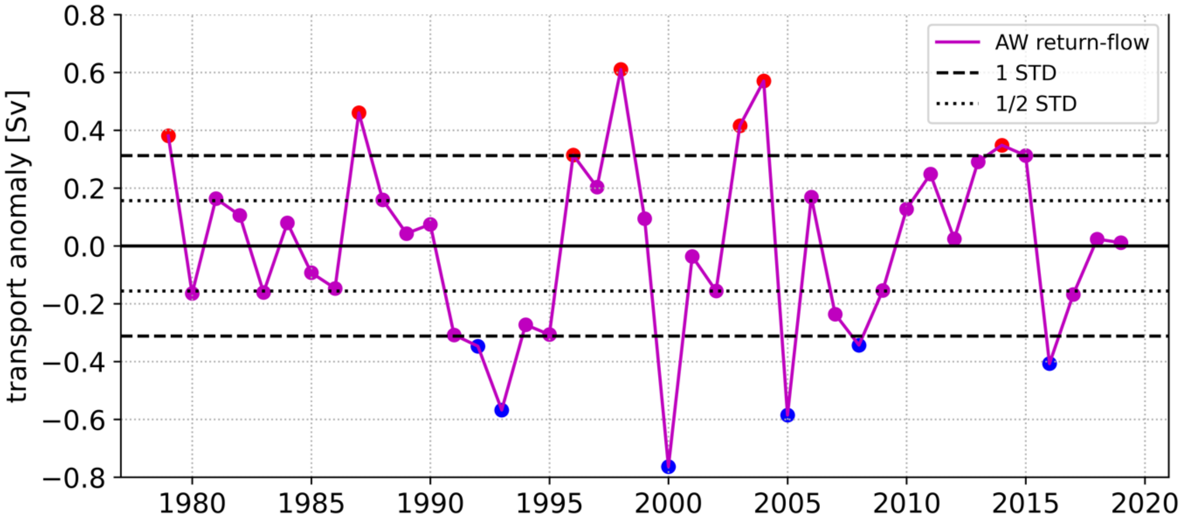

Fig. SI4: Volume Transport Anomalies of the BSO Return-Flow. Timeseries of detrended and anomalous volume transport of the BSO return-flow. Dashed and dotted lines indicate +-1 STD and +-0.5 STD. Red and blue markers indicate winters which are used in the composite analysis presented in Fig. 4.

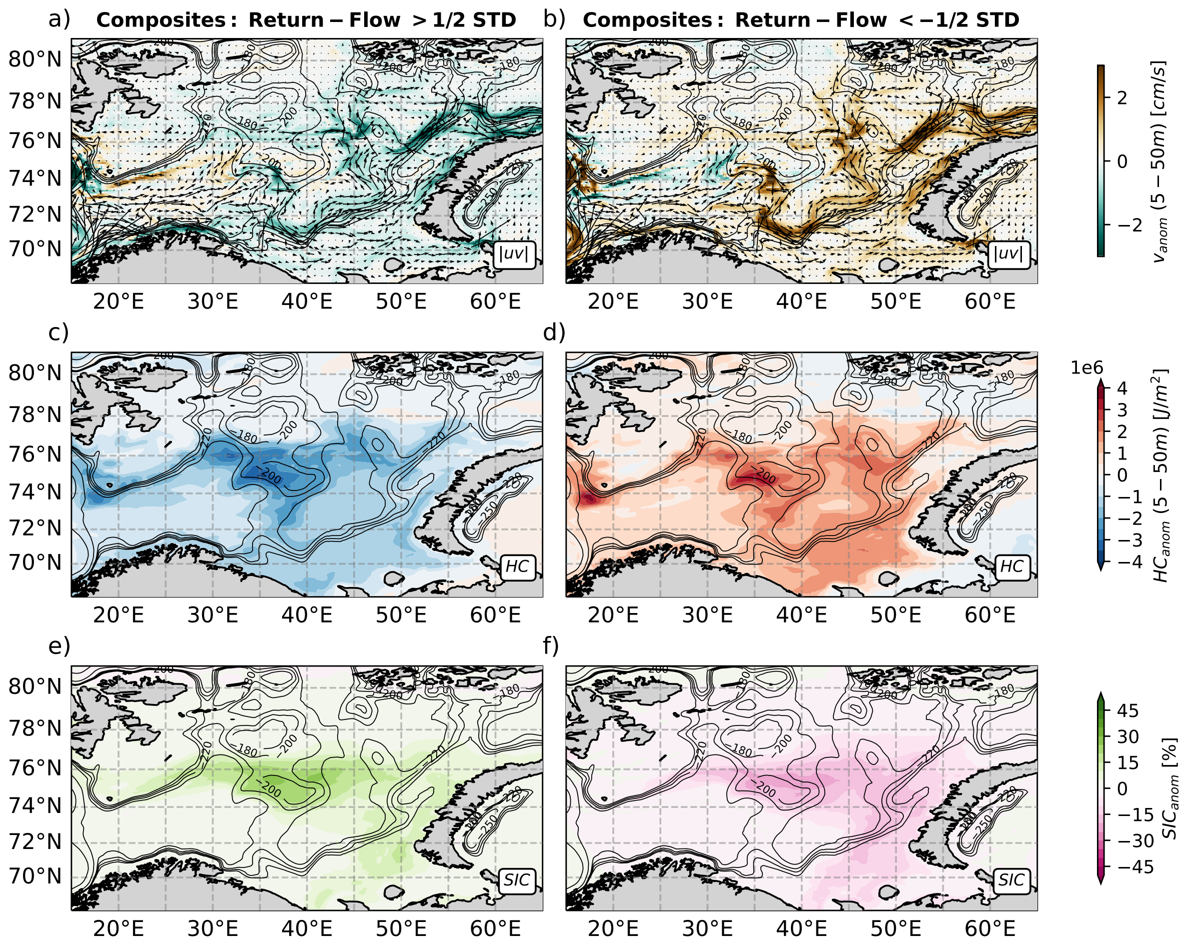

Fig. SI5: **Anomalous Atlantic Water transport through the Barents Sea.** *As in Fig. 4, but for years where the anomalous transport of the return-flow exceeds +- 0.5 STD (Fig. SI4).*
